# Can electronic search engines optimize screening of search results in systematic reviews: an empirical study

**DOI:** 10.1186/1471-2288-6-7

**Published:** 2006-02-24

**Authors:** Margaret Sampson, Nicholas J Barrowman, David Moher, Tammy J Clifford, Robert W Platt, Andra Morrison, Terry P Klassen, Li Zhang

**Affiliations:** 1 Chalmers Research Group, Children's Hospital of Eastern Ontario Research Institute, Ottawa, Canada; 2 Department of Pediatrics, Faculty of Medicine, University of Ottawa, Ottawa, Canada; 3 School of Mathematics and Statistics, Carleton University, Ottawa, Canada; 4 Canadian Coordinating Office for Health Technology Assessment, Ottawa, Canada; 5 Departments of Pediatrics and of Epidemiology and Biostatistics, McGill University, Montreal, QC, Canada; 6 Department of Pediatrics, University of Alberta, Edmonton, Canada; 7 Natural Sciences Library, University of Saskatchewan, Saskatoon, Canada

## Abstract

**Background:**

Most electronic search efforts directed at identifying primary studies for inclusion in systematic reviews rely on the optimal Boolean search features of search interfaces such as DIALOG^® ^and Ovid™. Our objective is to test the ability of an Ultraseek^® ^search engine to rank MEDLINE^® ^records of the included studies of Cochrane reviews within the top half of all the records retrieved by the Boolean MEDLINE search used by the reviewers.

**Methods:**

Collections were created using the MEDLINE bibliographic records of included and excluded studies listed in the review and all records retrieved by the MEDLINE search. Records were converted to individual HTML files. Collections of records were indexed and searched through a statistical search engine, Ultraseek, using review-specific search terms. Our data sources, systematic reviews published in the Cochrane library, were included if they reported using at least one phase of the Cochrane Highly Sensitive Search Strategy (HSSS), provided citations for both included and excluded studies and conducted a meta-analysis using a binary outcome measure. Reviews were selected if they yielded between 1000–6000 records when the MEDLINE search strategy was replicated.

**Results:**

Nine Cochrane reviews were included. Included studies within the Cochrane reviews were found within the first 500 retrieved studies more often than would be expected by chance. Across all reviews, recall of included studies into the top 500 was 0.70. There was no statistically significant difference in ranking when comparing included studies with just the subset of excluded studies listed as excluded in the published review.

**Conclusion:**

The relevance ranking provided by the search engine was better than expected by chance and shows promise for the preliminary evaluation of large results from Boolean searches. A statistical search engine does not appear to be able to make fine discriminations concerning the relevance of bibliographic records that have been pre-screened by systematic reviewers.

## Background

Systematic reviews in healthcare are designed to summarize and synthesize the existing totality of evidence about a clinical question using rigorous methods that help to safeguard the review from bias. Ideally, the evidence-gathering stage requires preparing as broad a search 'statement' as possible, and then adjusting the electronic search parameters to capture just the right kind of evidence to be useful without overburdening reviewers. More than three quarters of the studies included in systematic reviews are identified through electronic bibliographic databases that are searched using Boolean logic [1-5]. Boolean searches allow for complex query formulation, and are thus well suited to the exhaustive searching necessary for systematic reviews, but they allow no adjustment of threshold for retrieval based on probability or relevance [6]. Bibliographic records, often numbering in the thousands, require further evaluation to determine if they are in fact relevant to the review- a time intensive routine for clinical experts and other reviewers.

Search engines can rank search results by relevance based on where in the document the keywords appear, and how often. Using such a search engine to assign relevance to the large result set of a comprehensive Boolean search could reduce the amount of material requiring expert attention, if the documents that meet the strict eligibility criteria of the review are highly ranked.

Typically, the manual review of the results of Boolean electronic searches is often done in a two-stage process. In the first stage, reviewers may work from the bibliographic record, or document title, and screen out those that are obviously irrelevant to the review. In the second stage, reviewers usually obtain the full articles associated with remaining records and then decide eligibility based on the complete report, rather than on the more limited information available at the first stage of screening.

We evaluated a two-step approach consisting of a comprehensive Boolean search followed by automated relevance ranking for eligible systematic reviews from the Cochrane Library (Cochrane reviews henceforth) to explore the feasibility of such an approach.

## Methods

### Selection of systematic reviews

We sought Cochrane reviews that used at least one phase of the HSSS [7] to identify randomized controlled trials (RCTs) in MEDLINE. To be eligible, each Cochrane review must also i) have reported the citations for included and excluded studies and ii) have been a review of RCTs or quasiRCTs. The Cochrane Database of Systematic Reviews (CDSR) 3^rd ^Quarter 2002 was searched through the Ovid interface using the search string *(hsss or highly sensitive search).tw*. to identify potential studies. Two reviewers assessed each systematic review against the eligibility criteria and resolved any conflicts through consultation. SRS™ was used for all screening and data extraction. SRS is a web-based platform for conducting systematic reviews [8].

The size of the MEDLINE retrieval was determined by replicating and running the MEDLINE search strategy. Cochrane reviews with a MEDLINE retrieval of 1000–6000 records were selected for testing the performance of the Ultraseek search engine ranking.

### Data collection

One librarian extracted descriptive data about the eligible reviews. The following elements were recorded: the number of included studies and the number of excluded studies cited in the review; the number of included studies indexed in MEDLINE; the number of excluded studies indexed in MEDLINE; date of the MEDLINE search reported in the body of the review; level of detail in which the search was reported; phases of the HSSS used; searching techniques employed (such as thesaurus terms, term explosion, free text terms, truncation, adjacency operators); and restrictions such as date, language of publication, age groups or methodological filters. Electronic databases searched as well as other sources used (such as checking reference lists or contacting authors or manufacturers) were also recorded.

A known-item search was undertaken in MEDLINE for each included and excluded study listed in the review. A single librarian (MS) completed the searching using the Ovid interface for MEDLINE 1966-April 2003. The indexing status of each study was recorded as indexed or not indexed, and for each review, the set of included studies was aggregated using OR statements, as was a set of excluded studies. Each set was downloaded for subsequent analysis.

The Ovid bibliographic records for all studies retrieved from MEDLINE by the replicated search were also downloaded. When the review reported the size of the MEDLINE retrieval, it was compared to ours to validate the replication. Where our search result was smaller than that reported in the review, it was excluded as irreproducible.

### Search engine configuration

Produced by Verity, Ultraseek was originally a successful web search engine and is now focused on helping businesses manage their digital information [9]. The Ultraseek search engine (Version 5.0) was selected on the basis of its ability to deal with meta-data and assign weights to various fields. As we were dealing with indexed records and indexers have the benefit of access to the full text of the document, we anticipated that relevance ranking could be optimized by assigning greatest weight to terms appearing in indexing fields, intermediate weight to terms appearing in the title field, and lowest weight to terms appearing in the abstract field, following Hutchinson [10]. By comparison, in Boolean searching, each condition in the search is assigned a weight of 1 if present (i.e., the item is retrieved), and 0 if absent (i.e., the item is not retrieved).

The Ultraseek search engine indexes "collections". The bibliographic records associated with each systematic review were treated as a collection. Bibliographic records were downloaded from MEDLINE into Reference Manager databases, and tagged according to their inclusion status in the review. A Reference Manager output format was created to write each record with HTML tags. Three sets of fields were written as meta-data – MeSH headings, title and abstract (See sample record, Appendix 1). A Perl script was used to separate the HTML tagged bibliographic records into individual files. File names encoded the ID number of the review, whether the record was included or excluded from the review, and the reference ID number within that review, in the form http://10included3.html. Thus the collection consisted of the bibliographic records re-written as HTML files tagged with meta-data. The search engine was installed on a laptop computer where the collections resided. The search engine indexed all records in the collection. When a search was run against the collection, the number of items with relevance greater than zero was returned, along with list of up to the first 500 relevant items, sorted by relevance.

The Ultraseek search engine was configured to provide weights to the meta-data fields – index terms, title and description (abstract). When the weights given to the meta-data fields were varied in preliminary testing the relevance scores changed, but not the order of items, which was the variable of interest. Thus, the search engine was configured with all elements equally weighted, and the collections were indexed.

### Search terms

For each eligible review, one member of the research team (MS) identified subject terms to be entered into the Ultraseek search. In exploratory work, it became apparent that the number of tied relevance scores depended largely on the number of terms entered. Thus we decided to standardize our Ultraseek searches at 7 terms, the minimum number that seemed to reduce ties to a workable number.

We also established that the order in which terms were entered influenced the final relevance score. Terms were entered on the basis of perceived importance (see Table 3 for examples). A final eighth term, "random*" was included in each search. The asterisk is the truncation symbol used with Ultraseek.

Terms were selected to describe the topic of the review, focusing usually on the invention, but in some cases on the population. A number of reviews studied a constellation of interventions for a single condition, such as interventions for warts [11]. In those cases, the interventions may have been determined by reviewers *post-hoc*, so terms focused on the condition – warts – rather than on any interventions in the review. When the reviewers reported challenges in study identification, for instance, identifying injuries caused by distance running, versus running in other contexts, such as playing soccer [12], we attempted to address that difficulty in the selected terms.

Once the terms were defined, they were entered into the search box of the basic interface of the Ultraseek search engine. Terms were entered in lowercase text and truncated, and each collection was searched. Search outputs were saved for subsequent analysis.

### Analysis

We examined i) the rankings of included studies within a collection comprising the entire MEDLINE retrieval and ii) the ranking of included studies where only the studies listed as included or excluded in the Cochrane report comprised the collection. As we were concerned that the search engine might be optimized to place highly relevant items in the top few items with less exact ranking further back in the pack[13], we also examined the precision of the top 10 rankings [14].

When testing the initial MEDLINE retrieval, recall was determined by considering the proportion of included studies ranking within the top 500. We compared the proportion falling within the top 500, based on their relevance rank, with the proportion expected if ranking was random. When testing listed included and excluded studies, the rankings were analyzed with a Wilcoxon rank sum test using SAS for exact permutations, in order to best handle the small data sets and frequent ties [15].

## Results

### Eligible studies

**Figure 1:**
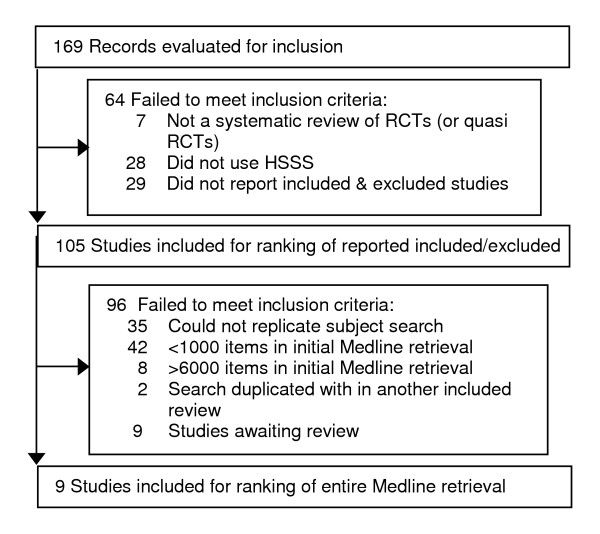
QUOROM diagram.

One hundred and sixty nine Cochrane reviews were retrieved and screened for eligibility. Sixty-four of them were excluded either because they do not use any phase of the HSSS or they did not report the citations of included and excluded trials. The remaining 105 reviews met our inclusion criteria (Figure 1) and formed the basis of our study sample. We were able to replicate the subject search for 61 of these reviews. Ten of them had between 1000 and 6000 records in the initial MEDLINE retrieval, and these were selected for replication of the MEDLINE retrieval. One review was excluded because our retrieval was much smaller than the initial MEDLINE retrieval reported by the authors [16]. Thus, we created collections that approximately replicated the initial MEDLINE retrieval for nine reviews [11,12,17-23]. Characteristics of these reviews are shown in Table 1.

**Table 1:** Characteristics of the 9 included reviews

Characteristic	N
Year of publication or substantive update	
2002	1
2001	1
2000	3
1999	1
1998	2
1997	1
	
Focus of the review	
Treatment	7
Prevention	1
Diagnosis	1
	
Study designs included	
RCT only	5
RCT and quasi-RCT	3
RCT and other controlled trials	1
	
Meta-analysis performed	7
	
Country of first author	
UK	4
Canada	2
Finland	1
Hong Kong	1
USA	1
	
Cochrane Review Group	
Musculoskeletal Injuries	2
Back	1
Eyes and Vision	1
Hypertension	1
Prostatic Diseases and Urologic Cancers	1
Skin	1
Stroke	1
Upper GI and Pancreatic Diseases	1

### Results of the full search replication of 9 reviews

We considered an item to be recalled if it appeared in the top 500, the number of items displayed by the search engine. Recall of included studies ranged from 0.35 to 1.00 (case by case results are shown in Table 2). Perfect recall was achieved in 3 of the 9 cases. We judged performance of the ranking to be good (0.80 or higher) in 6 of 9 cases.

**Figure 2:**
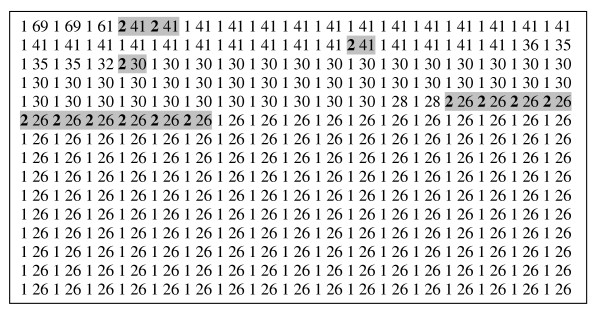
The review has 21 included studies. The MEDLINE search retrieved 1486 records. Ultraseek ranked 726 of the records as relevant and displayed the 500 with highest relevance scores. Inclusion status (1 = not included, 2 = included) is indicated, followed by the relevance score. The positions of records of included studies are highlighted.

An example of the result of ranking a collection is shown in Figure 2.

**Table 2:** Performance of the first ranking attempt for each review

Review	N of records retrieved from MEDLINE (d)	N of included studies ranked in the top 500 (d)	N of included indexed in MEDLINE (d)	Proportion of records selected by the search engine (p = a/b)	Recall (q = c/d)	p-value
Gibbs [11]	5743	11	27	0.09	0.41	<0.001
Yeung [12]	4996	6	11	0.10	0.55	<0.001
Smeeth [17]	3119	4	5	0.16	0.80	0.003
Towheed [18]	1556	6	17	0.32	0.35	0.80
Shelley [19]	1486	5	5	0.34	1.00	0.004
Karjalainen [20]	1244	2	2	0.40	1.00	0.16
Malthaner [21]	2321	6	6	0.22	1.00	<0.001
Bowen [22]	4629	12	14	0.11	0.86	<0.001
Mulrow [23]	1405	36	39	0.36	0.92	<0.001
Overall	26499	88	136	0.17	0.70	

Suppose there are *b *records in the initial retrieval. Suppose the top *a *(here we consider *a *= 500) of these records are selected by the search engine, i.e. a proportion *p *= *a*/*b*. Suppose further that this subset includes *c *of the *d *relevant records from the initial retrieval, i.e. a proportion *q *= *c*/*d*. If the search engine performs no better than would be expected by chance, then we would expect *q *= *p*. For each systematic review, we treated *c *as a binomial random variable with denominator *d*, and conducted a two-sided exact binomial test of the hypothesis that the expected value of *q *was equal to *p*.

**Table 3:** Comparison of first and second ranking attempt

Review	Attempt	Terms	Included in Top 500	Recall
Gibbs [11]	1	non-genital "human papilloma virus" hpv viral topical local cyrotherapy random*	11/27	0.41
	2	topical placebo* viral follow-up treatment* verru* wart* random*	18/27	0.67
Yeung [12]	1	run* distance prevent* reduc* footwear brace orthos* orthotic* random*	6/11	0.55
	2	prevent* tendon* knee ankle foot* hip injur* random*	6/11	0.55
Towheed [18]	1	osteoarthritis "global assessment" "range of motion" function* adverse toxicity hip random*	6/17	0.35
	2	placebo pain analgesic anti-inflammatory nsaid* hip osteoarthrit* random*	8/17	0.47

**Table 4:** Recall into the top 10

Review Number	N of records retrieved from MEDLINE	N of included studies ranked in the top 10	N of included indexed in MEDLINE	Recall
Gibbs [11]	5743	0	27	0.00
Yeung [12]	4996	1	11	0.09
Smeeth [17]	3119	2	5	0.40
Towheed [18]	1556	0	17	0.00
Shelley [19]	1486	0	5	0.00
Karjalainen [20]	1244	0	2	0.00
Malthaner [21]	2321	2	6	0.33
Bowen [22]	4629	2	14	0.14
Mulrow [23]	1405	0	39	0.00
Total	26499	7	136	0.06

The ranking performance was significantly better than would have been expected by chance for 7 of the 9 reviews (although perfect recall was achieved in one of the non-significant instances).

For three reviews where the Ultraseek ranking performed poorly, we attempted another search, using a different set of terms. Terms and performance on each of the two attempts are reported in Table 3. The second attempts did not appear substantially better than the first attempts.

### Results of searching included and excluded studies

There was no statistically significant difference between the rankings of studies listed as included versus studies listed as excluded (analogous to studies excluded at a second stage of screening studies). Further, no obvious pattern between the included and excluded studies could be discerned through visual inspection (see for example, Figure 2).

## Discussion

The number of published systematic reviews has increased 500 fold over the last decade or so [24]. This increase can be attributed, in part, to their growing importance within the healthcare community. Beyond their traditional role of accumulating the totality of evidence regarding the effectiveness of a variety of interventions, they are being increasingly used by healthcare policy analysts and others to inform decision-making across a very broad range of issues. Systematic reviewers will need to become ever more innovative if reviews are to maintain the timeliness necessary for decision makers [25,26]. One important way this can be achieved is by developing robust and scientifically valid methods to enhance the speed in which reviews can be completed. Part of this efficiency is likely to come through decreasing the time taken to filter out articles that are irrelevant to the question under consideration. The relevance ranking searching approach has gained broad support and we investigated whether it might be a useful approach to aid in the conduct of systematic reviews.

Our results support the feasibility of relevance ranking Boolean search result sets from systematic reviews. A previous effort at relevance ranking search results concluded that ranking techniques employed by Internet search engines do not facilitate effective retrieval [27]. The clearly defined search questions[28] or the highly refined assessment of relevance [29] used in systematic reviews, such as the Cochrane reviews included in our study, may account for the greater utility shown here.

Sorting the entire initial MEDLINE retrieval showed promise. Working with sets of between 1000 and 6000 records, we were able to select search terms that could rank most included studies in the top 500. We do interpret this as showing some support for a two zone pre-processing model in which the reviewers' screening load is lightened by removing the items receiving the lowest relevance ranks. A limitation was the dependence on term selection and even the ordering of terms, thus performance could depend on operator characteristics. Further, using a limited number of terms as keywords for retrieval prevents the formation of elaborate queries [30], and we experienced difficulty representing some of the topics of the reviews. Other approaches, such as natural language queries, have been explored as alternatives to Boolean retrieval of medical literature [31,32].

The approach used here could not distinguish between the included and excluded study lists of Cochrane reviews. We interpret this to mean that when differences between included and excluded studies are slight, (i.e., presentation in the Cochrane excluded study list is warranted) a relevance-ranking search engine will have difficulty distinguishing relevant from irrelevant items on the basis of the information contained in the bibliographic record.

The approach tested here is interesting as a proof of concept. Truncating the search result at 500 records would have resulted in great efficiencies, reducing the number of records to be reviewed from 26499 to 4500 across the nine reviews, but at the expense of losing 30% of relevant studies – few reviewers would likely accept such a trade-off.

Incorporating additional information into the rankings might facilitate any future exploration of using search engines to reduce the time taken to conduct a systematic review. There are at least four potential sources of additional information. First, giving increased weight to information in the title and index terms is likely to improve performance; we were not able to do this through meta-data weightings. A second source of information to improve accuracy would be the full document. Our results were based on the information contained in a document surrogate – the bibliographic record. A full text approach will become practical as a larger proportion of articles considered for inclusion in systematic reviews become available electronically.

A third source of additional information would be the prior decisions of the reviewers. Novel techniques developed by de Bruijn and Martin [33] have been effective in discerning patterns in the decision making of reviewers to determine relevance of bibliographic citations. A formal test of such techniques would be extremely interesting. Finally, a fourth type of information could be corroborating evidence through citation in a prior review on the same topic or a paper already deemed relevant. Science Citation Index allows both subject searching and citation searching, and other search interfaces are increasingly providing this integration[34,35].

Rankings could be used in several ways. Our finding that included studies receive higher than chance rankings but that the included *vs*. excluded lists could not be distinguished points to a role for ranking at the broad screening stage of the review. We did not see a strong enough concentration of relevant records in the top 10 to suggest that some records were so clearly relevant that they should be promoted into the review without manual examination. A useful step would have been to establish a lower cut-off point, below which we could be confident that no relevant studies would fall. This would have permitted us to exclude the lowest ranking (including non-ranked) records without subjecting them to any manual review, thereby gaining efficiency. The search engine selected for the experiment did not enable us to examine any but the first 500 hits.

## Conclusion

Relevance ranking of Boolean search results is technically feasible using a commercial search engine under academic license to index and search collections built through a simple procedure to render Reference Manager^® ^records into HTML format. Ranking holds promise and future research is warranted to explore methods that incorporate more information into the relevance ranking process. A system reliably placing the relevant records within a smaller ranked set derived from the Boolean retrieval can speed the formation of the evidence base for systematic reviews in healthcare.

## Appendix 1

Sample bibliographic record showing HTML markup and meta data tags

<html>

<head>

<meta name="author" content="Fabacher, D., Josephson, K., Pietruszka, F., Linderborn, K., Morley, J. E., and Rubenstein, L. Z.">

<meta name="title" content="An in-home preventive assessment program for independent older adults: a randomized controlled trial. -see comments.-">

<meta name="subjects" content="Activities of Daily Living;Aged;Comparative Study;Female;Follow-Up Studies;Geriatric Assessment;Health Status;Home Care Services;Human;Male;Non-U.S.Gov't;Patient Compliance;Preventive Health Services;og -Organization & Administration-;Support;United States;Veterans;Voluntary Workers">

<meta name="Abstract" content="OBJECTIVE: To evaluate the effectiveness of in-home geriatric assessments as a means of providing preventive health care and improving health and functional status of ... truncated for illustration ... aspects of health and function">

<!-- SR ID = 10-->

<!-- inclusions status = excluded-->

<!-- Ph1 or Ph1 = yes-->

<!-- NBSS = yes-->

<!-- Ph3 only = no-->

<title>An in-home preventive assessment program for independent older adults: a randomized controlled trial. -see comments.-</title>

</head>

<body>

<br>Ref ID = 3<br>

<br>Fabacher, D., Josephson, K., Pietruszka, F., Linderborn, K., Morley, J. E., and Rubenstein, L. Z. An in-home preventive assessment program for independent older adults: a randomized controlled trial. -see comments.-.Journal of the American Geriatrics Society 1994;42(6):630–638.<br>

<P>MeSH Subject Headings:

<br>Activities of Daily Living;Aged;Comparative Study;Female;Follow-Up Studies;Geriatric Assessment;Health Status;Home Care Services;Human;Male;Non-U.S.Gov't;Patient Compliance;Preventive Health Services;og -Organization & Administration-;Support;United States;Veterans;Voluntary Workers

<P>Abstract:

<br>OBJECTIVE: To evaluate the effectiveness of in-home geriatric assessments as a means of providing preventive health care and improving health and functional status of ... truncated for illustraton ... aspects of health and function<br>

</body>

</html>

## Competing interests

The author(s) declare that they have no competing interests.

## Authors' contributions

*MS *conceptualized the project, screened records for eligibility, undertook data collection and analysis, created the Reference Manager output format, selected search terms, prepared the first draft of the manuscript, and participated in all revisions. *NJB *obtained funding for the project, designed the statistical analysis and created the Perl scripts, and participated in the drafting and revision of the manuscript. *DM *obtained funding for the project, advised on the design and conduct of the experiments and participated in all revisions of the manuscript. *TJC *acted as project leader for the grant, advised on the design and conduct of the experiments and participated in all revisions of the manuscript. *RWP *obtained funding for the project, advised on the statistical analysis, the design and conduct of the experiments and participated in all revisions of the manuscript. *AM *screened records for eligibility, undertook data collection, and participated in all revisions of the manuscript. *TK *obtained funding for the project, the design and conduct of the experiments and participated in all revisions of the manuscript. *LZ *configured the search engine, replicated the searches, undertook known item searching, built and indexed the collections, created the datasets, and participated in all revisions of the manuscript.

All authors reviewed and approved the final manuscript.

## Pre-publication history

The pre-publication history for this paper can be accessed here:


